# Assessment of variation in the alberta context tool: the contribution of unit level contextual factors and specialty in Canadian pediatric acute care settings

**DOI:** 10.1186/1472-6963-11-251

**Published:** 2011-10-04

**Authors:** Carole A Estabrooks, Janet E Squires, Alison M Hutchinson, Shannon Scott, Greta G Cummings, Sung Hyun Kang, William K Midodzi, Bonnie Stevens

**Affiliations:** 1 Faculty of Nursing, University of Alberta, Edmonton, Canada; 2 Clinical Epidemiology Program, Ottawa Hospital Research Institute, Ottawa, Canada; 3 Faculty of Nursing, Deakin University, and Cabrini-Deakin Centre for Nursing Research, Cabrini Institute, Cabrini Health, Melbourne, Australia; 4 Department of Medicine, Memorial University of Newfoundland, St. John's, Canada; 5 The Hospital for Sick Children and the Lawrence S. Bloomberg Faculty of Nursing, University of Toronto, Toronto, Canada

## Abstract

**Background:**

There are few validated measures of organizational context and none that we located are parsimonious and address modifiable characteristics of context. The *Alberta Context Tool *(ACT) was developed to meet this need. The instrument assesses 8 dimensions of context, which comprise 10 concepts. The purpose of this paper is to report evidence to further the validity argument for ACT. The specific objectives of this paper are to: (1) examine the extent to which the 10 ACT concepts discriminate between patient care units and (2) identify variables that significantly contribute to between-unit variation for each of the 10 concepts.

**Methods:**

859 professional nurses (844 valid responses) working in medical, surgical and critical care units of 8 Canadian pediatric hospitals completed the ACT. A random intercept, fixed effects hierarchical linear modeling (HLM) strategy was used to quantify and explain variance in the 10 ACT concepts to establish the ACT's ability to discriminate between units. We ran 40 models (a series of 4 models for each of the 10 concepts) in which we systematically assessed the unique contribution (i.e., error variance reduction) of different variables to between-unit variation. First, we constructed a null model in which we quantified the variance overall, in each of the concepts. Then we controlled for the contribution of individual level variables (Model 1). In Model 2, we assessed the contribution of practice specialty (medical, surgical, critical care) to variation since it was central to construction of the sampling frame for the study. Finally, we assessed the contribution of additional unit level variables (Model 3).

**Results:**

The null model (unadjusted baseline HLM model) established that there was significant variation between units in each of the 10 ACT concepts (i.e., discrimination between units). When we controlled for individual characteristics, significant variation in the 10 concepts remained. Assessment of the contribution of specialty to between-unit variation enabled us to explain more variance (1.19% to 16.73%) in 6 of the 10 ACT concepts. Finally, when we assessed the unique contribution of the unit level variables available to us, we were able to explain additional variance (15.91% to 73.25%) in 7 of the 10 ACT concepts.

**Conclusion:**

The findings reported here represent the third published argument for validity of the ACT and adds to the evidence supporting its use to discriminate patient care units by all 10 contextual factors. We found evidence of relationships between a variety of individual and unit-level variables that explained much of this between-unit variation for each of the 10 ACT concepts. Future research will include examination of the relationships between the ACT's contextual factors and research utilization by nurses and ultimately the relationships between context, research utilization, and outcomes for patients.

## Background

Implementation science is the investigation of methods, interventions, and variables that shape the use of research findings in practice, i.e., research utilization. Research demonstrates that contextual factors, i.e., the work setting, consistently moderate strategies to move research into clinical practice [1-3]. Therefore, understanding contextual factors is important to advancing the science of research utilization [4-7]. However, investigation is needed to understand what factors influence context and how context in turn shapes the use of research findings in practice. A better understanding of both of these processes will in turn inform the development and evaluation of interventions to increase research use by healthcare providers, the goal of which is improved patient and organizational (system) outcomes [8,9]. Integral to this goal is the ability to assess and quantify context [10,11]. The Alberta Context Tool (ACT) was developed to meet this goal.

### The Alberta Context Tool (ACT)

The ACT is a parsimonious survey designed to measure organizational context in complex healthcare settings. It is administered at the level of the individual healthcare provider to elicit their perception of context at the patient care unit and/or organizational (hospital) level, depending on the context of care delivery. For nurses, this level is predominantly at the patient care unit.

Three principles guided the development of the ACT: (1) substantive theory, (2) brevity (ability to complete the instrument in 10 minutes or less), and (3) modifiability (focus on researchable elements of context which are amendable to change). We used the *Promoting Action on Research Implementation in Health Services *(PARiHS) framework [12] to conceptualize organizational context. Where the framework did not provide direction, we operationalized concepts from related literature (e.g., [13-16]). The PARiHS framework has three core elements - evidence, facilitation and context - which are considered essential to the successful implementation of research into practice [10,12,17]. In this framework, context is understood to be the environment or setting where research is to be implemented, and is proposed to have three discrete dimensions: culture, leadership and evaluation [12]. Culture is defined as "the forces at work, which give the physical environment a character and feel" [17] (p.97). Leadership is defined as the "nature of human relationships" [17] (p.98). Effective leadership, in this framework, is conceptualized to give rise to clear roles, effective teamwork and organizational structures, and the involvement of organizational members in decision making and learning. Evaluation, in the PARiHS framework, refers to feedback mechanisms (individual and system level), sources, and/or methods for evaluation [12].

The ACT survey consists of a series of items representing 8 dimensions that are comprised of 10 contextual concepts: (1) leadership, (2) culture, (3) evaluation, (4) social capital, (5) structural and electronic resources, (6) formal interactions, (7) informal interactions, (8) organizational slack - staffing, (9) organizational slack - space, and (10) organizational slack - time. Definitions and sample items of the eight context dimensions are listed in Table 1. The survey exists in three versions (adult care, pediatric care, and long-term care), each with multiple forms (nurses, allied healthcare providers, practice specialists, physicians, and managers). The pediatric nurse version, reported in this paper, consists of 56 items and underwent initial assessment for reliability and validity using data from a national, multi-site study with pediatric nurse professionals [18]. In that report, a principal components analysis (PCA) indicating a 13-factor solution (accounting for 59.26% of the variance in 'organizational context') was reported. Bivariate associations between research utilization levels and the majority of ACT factors as defined by the PCA were statistically significant at the 5% level. Each ACT factor also showed a trend of increasing mean scores ranging from the lowest level to the highest level of research use, further supporting construct validity. The instrument also demonstrated adequate internal consistency reliability with Cronbach's alpha coefficients ranging from a low of 0.54 to a high of 0.91 for the 13 factors [18].

**Table 1:** Dimensions of the ACT

Concept	Definition	Sample item
Leadership^1^	The actions of formal leaders in an organization (unit) to influence change and excellence in practice, items generally reflect emotionally intelligent leadership	The leader calmly handles stressful situations

Culture^1^	The way that "we do things" in our organizations and work units; items generally reflect a supportive work culture	My organization effectively balances best practice and productivity

Evaluation^1^	The process of using data to assess group/team performance and to achieve outcomes in organizations or units (i.e., evaluation)	Our team routinely monitors our performance with respect to the action plans

Social Capital^1^	The stock of active connections among people. These connections are of three types: bonding, bridging, and linking	People in the group share information with others in the group

Informal Interactions^2^	Informal exchanges that occur between individuals working within an organization (unit) that can promote the transfer of knowledge	How often do you interact with people in the following roles or positions?; - Someone who *champions *research and its use in practice

Formal Interactions^2^	Formal exchanges that occur between individuals working within an organization (unit) through scheduled activities that can promote the transfer of knowledge	How often do these activities occur?; - Team meetings

Structural/Electronic Resources^3^	The structural and electronic elements of an organization (unit) that facilitate the ability to assess and use knowledge	How often do you use/attend the following?; - A library

Organizational Slack	The cushion of actual or potential resources which allows an organization (unit) to adapt successfully to internal pressures for adjustments or to external pressures for changes	
Staffing^1^		Enough staff to deliver quality care
Time^1^		Time to do something extra for patients
Space^1^		Use of designated space

^1^= Scale: 1-strongly disagree; 2-disagree; 3-neither agree or disagree; 4-agree; 5-strongly agree; ^2^= Scale: 1-never; 2-rarely; 3-ocasionally; 4-frequently; 5-almost always;

^3^= Scale: 1-never; 2-rarely; 3-ocasionally; 4-frequently; 5-almost always; 6- not accessible

In a subsequent validity assessment of the ACT [19], completed on responses obtained from healthcare aides (i.e., unregulated nursing care providers) in residential long-term care settings (i.e., nursing homes), we assessed advanced aspects of validity using the Standards for Educational and Psychological Testing (the *Standards*) validation framework, considered best practice in psychometrics [20]. The *Standards *identifies four sources of validity evidence, all of which contribute to construct validity. The four sources are: content evidence (the extent to which the items represent the content domain of the concept), response processes evidence (how respondents interpret, process, and elaborate upon item content and whether this is in accordance with the concept being measured), internal structure evidence (relationships between the items within a concept), and relations to other variables evidence (relationships between the concept of interest and external variables with which it is expected and not expected to be related) [20]. In the latter validation paper conducted with healthcare aides in nursing homes, we extended our initial validity assessment and examined advanced aspects of internal structure validity evidence (e.g., confirmatory factor analyses) as well as additional relations with other variables validity testing. The overall pattern of the data (assessed in the confirmatory factor analyses) was consistent with the hypothesized structure of the ACT. Additionally, eight of the ten ACT concepts were related, at statistically significant levels, to instrumental research utilization, supporting the construct validity of the ACT [19].

### Patient Care Units as Microsystems

The microsystems literature emphasizes the importance of directing system improvement strategies at the level of clinical (patient care) units. Its proponents argue that these units where care delivery occurs are the essential building blocks or functional units of the organization [21-25]. The clinical unit represents a complex and dynamic system, characterized by interaction between various elements or features (such as leadership, culture, personnel and information) in the process of care delivery [21]. The term 'unit' implies a discrete entity, the margins of which, typically, are defined by geographic limits and the practice specialty [26]. According to the microsystems literature however, "the clinical unit has a semipermeable boundary that mediates relationships with patients and with many support services and external microsystems" [21] (p. 476).

Organizations or macrosystems are comprised of mesosystems such as programs and centers, which, in turn, consist of these connected and interrelated microsystems or units. Nursing care tends to be organized at the level of the clinical unit [4]. Thus, individual patients receive care in clinical units (microsystems) that are embedded within departments, services or programs, which are integrated to form healthcare organizations [27]. Targeting improvement strategies at the level of the functional unit, therefore, has the potential to transform healthcare systems and the patient care experience [21]. Research examining clinical microsystems indicates that high performing units are associated with better patient outcomes [21].

The microsystems literature acknowledges that the effectiveness of healthcare providers is, in part, mediated by the context or environment in which they work [22]. Thus, knowledge of unit context is essential to the development of interventions to optimize care. The microsystems approach aims to understand the context of care delivery, to design systems that enable and support healthcare providers to deliver care consistent with best practice (research) and, ultimately to ensure that patients receive safe, high quality care [22]. The work of Sales et al. [26] reinforces the importance of studying units as individual entities. They found that because of heterogeneity between units, aggregation of nurse data above the level of unit produced biased results and poor estimates of associations with quality measures. This highlights the importance of determining unit-level estimates and identifying variation between microsystems.

The purpose of this paper is to report evidence to further a validity argument for the ACT (which measures context) when used in pediatric settings with professional nurses to capture unit-level context scores. Specifically, we (1) examined the extent to which the 10 ACT concepts discriminate between patient care units, and (2) identified variables that contribute to explaining the between-unit level variation in each of the 10 concepts. While assessment of between-unit discrimination and variance is not a traditional form of validity testing, it is essential to understanding the construct validity of instruments like the ACT that collect data at the individual (respondent) level with the purpose of aggregating those responses to obtain higher (e.g., unit) level estimates.

## Methods

### Design, Sample, and Data Collection

We used a cross-sectional survey design. Thirty-two patient care units in eight pediatric hospitals across Canada provided the sampling pool for the ACT's administration. The 32 units were distributed between medical units, surgical units and critical care units (neonatal and pediatric intensive care). Five healthcare professional groups were eligible to participate: (1) nurses, (2) physicians, (3) allied healthcare professionals, (4) clinical specialists (e.g., educators), and (5) managers. Inclusion and exclusion criteria for the professional subgroups are presented in Additional File 1. For psychometric testing reasons, we wanted a homogeneous sample and therefore conducted the analysis reported here on the largest group of respondents - nurses (which accounted for 67% of the total sample). Data were collected using an online survey and compiled in a centralized database at the core site for the study. Eligible participants were provided with a survey package containing a letter introducing the study, and a business card providing a Uniform Resource Locator (URL) and unique password to access the survey online.

Ethical approvals for this study were obtained from the Health Research Ethics Boards of the appropriate universities, as well as, the hospital ethics review boards (where applicable) for all hospitals participating in the study.

### Measures

The analyses reported here use data from two data collection instruments: (1) the Translating Research on Pain in Children (TROPIC) Unit Profile Form, and (2) the TROPIC Survey (in which the ACT was embedded), both developed specifically by the research team for this study. The TROPIC Unit Profile Form consists of a series of questions about the structural and human resources available on each unit. Examples of items include: average length of patient stay and the number of nurses working on the unit. A research nurse at each site completed the form electronically; a training session preceded data collection. All data were then compiled together at a centralized data collection centre, at the core site for the study. The TROPIC survey was used to collect provider (staff)-level data. The survey is composed of a suite of survey instruments designed to measure: (1) organizational context, (2) research utilization, (3) staff outcomes (e.g., health status, job satisfaction), and (4) select other individual and organizational factors believed to influence research utilization and staff outcomes. The core of the TROPIC Survey is the ACT. Development of the ACT and the results of its initial psychometric assessment are summarized in the background section of this paper, with further details published in an earlier issue of this journal [18].

### Study Variables

#### Dependent variables

The dependent variables examined in this study were the 10 contextual concepts of the ACT (See Table 1). To obtain one score for all items within a concept, the individual items within each concept were averaged (culture, leadership, evaluation, social capital, organizational slack-staffing, organizational slack-time, organizational slack-space) or recoded as existing or not existing and then counted or summed (informal interactions, formal interactions, structural and electronic resources).

#### Independent variables

The independent variables included in our analyses are listed in Table 2. The research team selected these variables from those available on the TROPIC Unit Profile Form and the TROPIC survey based on current knowledge represented in the (organizational) context in healthcare literature. The independent variables were verified in a series of team meetings as being either at the individual-level (Level 1) or at the unit-level (Level 2).

**Table 2:** Descriptive Statistics for Individual and Unit-Level Variables by Specialty

Variables	Overall	Surgical	Medical	Critical Care	P-value^1^
***Individual level variables, N***	**844**	**199**	**329**	**316**	
Employment Status (N, %)					
Full-time	517 (61.8%)	120 (60.3%)	199 (60.5%)	198 (62.7%)	0.977
Part-time	295 (35.0%)	71 (35.7%)	117 (35.6%)	107 (33.9%)	
Casual	32 (3.8%)	8 (4.0%)	13 (4.0%)	11 (3.5%)	

Education (N, %)					
Diploma	305 (36.3%)	79 (39.9%)	102 (31.0%)	124 (39.6%)	0.029
Bachelor	522 (62.1%)	118 (59.6%)	218 (66.3%)	186 (59.4%)	
Masters or Higher	13 (1.5%)	1 (0.5%)	9 (2.7%)	3 (1.0%)	

Age (N, %)					
20-24 YEARS	84 (10.0%)	25 (12.6%)	40 (12.2%)	19 (6.0%)	0.000
25-29 YEARS	239 (28.3%)	60 (30.2%)	115 (35.0%)	64 (20.3%)	
30-34 YEARS	143 (16.9%)	28 (14.1%)	56 (17.0%)	59 (18.7%)	
35-39 YEARS	89 (10.5%)	22 (11.1%)	36 (10.9%)	31 (9.8%)	
40-44 YEARS	82 (9.7%)	15 (7.5%)	31 (9.4%)	36 (11.4%)	
45-49 YEARS	83 (9.8%)	12 (6.0%)	25 (7.6%)	46 (14.6%)	
50-54 YEARS	75 (8.9%)	22 (11.1%)	16 (4.9%)	37 (11.6%)	
55-59 YEARS	42 (5.0%)	14 (7.0%)	6 (1.8%)	22 (7.0%)	
60-64 YEARS	6 (0.7%)	1 (0.5%)	4 (1.2%)	1 (0.3%)	

MBI-Exhaustion (Mean, STD)	2.287 (1.249)	2.401 (1.308)	3.859 (0.853)	3.839 (0.813)	0.332

Adequate Orientation (Mean, STD)	3.953 (0.756)	2.240 (1.208)	3.961 (0.734)	3.939 (0.734)	0.107

Job Satisfaction (Mean, STD)	3.910 (0.774)	2.287 (1.249)	3.953 (0.756)	3.924 (0.789)	0.327

***Unit level variables, N***	**32**	**8**	**14**	**10**	
MBI-Cynicism (Mean, STD)	2.230 (0.687)	2.292 (0.718)	2.095 (0.712)	2.371 (0.664)	0.612

MBI-Efficacy (Mean, STD)	3.825 (0.711)	3.820 (0.693)	3.845 (0.824)	3.802 (0.618)	0.990

Years on Unit (Mean, STD)	7.734 (2.900)	7.108 (2.734)	6.425 (2.183)	10.068 (2.683)	0.004

French/Anglophone (N, %)					
French	4 (12.5%)	1 (12.5%)	2 (14.3%)	1 (10.0%)	0.952
English	28 (87.5%)	7 (87.5%)	12 (85.7%)	9 (90.0%)	

# of occupied unit beds (Mean, STD)	20.043 (10.069)	21.675 (4.862)	20.756 (10.037)	17.741 (13.279)	0.684

Support for innovative ideas (Mean, STD)	3.290 (0.288)	3.290 (0.281)	3.389 (0.197)	3.152 (0.363)	0.138
Proportion of baccalaureate or higher (Mean, STD)	71.666 (14.324)	68.913 (14.075)	77.282 (15.345)	66.007 (11.007)	0.134

^1 ^P-value for ANOVA or χ^2 ^-test (by specialty)

### Analytic Approach

#### Reliability and validity of aggregated data at the unit level

Aggregation of individual-level data to a higher (e.g., unit) level is an important methodological issue that has received minimal attention in health services research. While direct measurement of unit-level concepts (e.g., culture) is preferable, it is most often not possible. Therefore, in order to include unit-level estimates of these concepts in our statistical models, we need to obtain data on them from individuals and then aggregate these data to the higher (unit) level. One concern with aggregation is that as data are aggregated, less information will be carried-up to the higher level than is optimal. Therefore, the first step in our analysis was to examine the reliability and validity of all independent variables aggregated to the unit-level. We calculated four standard empirical aggregation indices for this assessment: (1) intraclass correlation 1, ICC(1); (2) intraclass correlation 2, ICC(2); (3) eta-squared, η^2^; and (4) omega-squared, ω^2^. One-way analysis of variance (ANOVA) was performed on each variable using the unit as the group variable. The source table from the one-way ANOVA was used to calculate the four standard aggregation indices.

ICC(1) is a measure of individual score variability about the subgroup mean. ICC(1) values theoretically can range from 0 to 1, with values of 0 indicating no perceptual agreement and values of 1 indicating perfect perceptual agreement among members within the same group. Therefore, values greater than 0 (0.10 is the accepted standard) indicate a degree of coherence among individuals about the mean values within each group (i.e., unit) [28]. James [29] examined ICC(1) values reported in applied psychological research studies to justify some degree of perceptual agreement among group members; values ranged from 0 to 0.5, with a median of 0.12. Others have reported similar values. For example, Bliese [30] and Vogus and Sutcliffe [31] reported that ICC(1) values in applied research typically fall between 0.05 to 0.20 and 0.5 to 0.30, respectively. ICC(2) is a measure of stability of aggregated data at the group level; values exceeding 0.60 justify aggregation [28]. η^2 ^and ω^2 ^are measures of validity, also known as measures of 'effect size' in ANOVA. An effect size is a measure of the strength of the relationship between two variables and thus, illustrates the magnitude of the relationship. η^2 ^denotes the proportion of variance in the individual variable (in each derived ACT concept) accounted for by group membership (e.g., by belonging to a specific nursing unit) [32]. This value is equivalent to the R-squared value obtained from a regression model, and where group sizes are large, to ICC(1) [30]. ω^2 ^measures the relative strength of aggregated data as an independent variable. It is also an estimate of the amount of variance in the dependent variable (e.g., in each derived ACT concept) accounted for by the independent variable (i.e., by group membership - belonging to a specific nursing unit) [33]. Larger values of η^2 ^and ω^2 ^indicate stronger effect sizes and relationships between variables. As a result, larger values of η^2 ^and ω^2 ^also indicate stronger 'relations to other variables' validity evidence (as described in the *Standards *validation framework) and thus, contribute to overall construct validity. Details on the methods for calculating each of these standard aggregation indices are located in our previous work [4,18,34,35].

There are multiple methods for calculating intraclass correlations (ICC). The two most widespread methods are from: (1) random coefficient (multi-level) models, calculated as ICC = unit-level variance/(unit-level variance + individual level variance), and (2) one-way random-effects ANOVA model, calculated as ICC(1) = (BMS - WMS)/(BMS + [K-1] WMS), where BMS = between mean square, WMS = within mean square, and K = the number of participants per group. At this stage of our analyses (which is preliminary to conducting the multi-level modeling) we were seeking statistical support for aggregating some individual variables to the unit-level before entering them into the models. Therefore, we chose to calculate ICC using the latter formula (from one-way random-effects ANOVA model). ICC using this model is referred to as ICC(1) [29,36,37], or ICC(1,1) [38]. The two methods of calculating ICC will produce, similar, but not identical, estimates (See Additional File 2). However, by running a one-way random-effects ANOVA model at this stage of our analysis, we were also able to calculate the remaining standard aggregation statistics (ICC(2), η^2^, and ω^2 ^described previously) in addition to the ICC(1). This allowed us to obtain a more thorough picture of the reliability and validity of our variables when aggregated to the unit-level.

#### Multi-level analysis

The data collected for this study had a natural hierarchical (or clustered) structure, that is, nurse respondents were nested within patient care units, which were nested within pediatric hospitals. Therefore, our main analysis consisted of a series of multilevel models. The multilevel analyses were conducted using two levels. Level 1 had individual (nurse) variables and Level 2 had unit-level variables. We were limited to two levels by sample size (that is, we did not have sufficient hospitals at the third level, n = 8 hospitals). We used hierarchical linear modeling (HLM) [39] to fit a series of multilevel models capable of quantifying the within-unit (Level 1) and between-unit (Level 2) variation among the 10 contextual concepts in the ACT. A detailed description of the application of two-level multilevel models in nursing organizational research is described elsewhere [40]. The modeling was done using SAS 9.2, MLwiN 2.12, and HLM 6.06.

##### Individual-level variables

Six individual-level variables were examined and controlled for in the analysis. They were: (1) education, (2) employment status, (3) age, (4) adequate orientation, (5) job satisfaction, and (6) burnout-emotional exhaustion. These factors were conceptualized as individual variables and analysed at Level 1. Each variable (with the exception of burnout-emotional exhaustion) was collected using a single item on the TROPIC survey. Burnout-emotional exhaustion is one of three subscales on the Maslach Burnout Inventory [41], which was embedded in the TROPIC Survey. The emotional exhaustion subscale consists of three items scored on a 7-point Likert-type scale (0-6); a mean of the three items derives an overall score. Higher scores indicate higher levels of burnout.

##### Unit-level variables

Eight unit-level variables were examined and controlled for in the analysis. They were: (1) burnout-cynicism, (2) burnout-efficacy, (3) experience (length of time) on the unit, (4) support for innovative ideas, (5) the proportion of nurses possessing a baccalaureate degree or higher, (6) language of survey completion (English or French), (7) practice specialty (medicine, surgery, critical care), and (8) the number of beds in the unit.

Burnout-cynicism and burnout-efficacy are the remaining two subscales of the Maslach Burnout Inventory [41]. Like the emotional exhaustion subscale discussed above, the cynicism and efficacy subscales also consist of three items, each scored on a 7-point Likert-type scale. An overall score is derived for each subscale by taking a mean of the three items; higher scores on cynicism and lower scores on efficacy equate with higher burnout. These two burnout subscales were conceptualized as unit-level variables on the basis of a standard aggregation statistic, ICC(1). ICC(1) values for both subscales exceeded 0.1 (values were 0.201 and 0.297 for the cynicism and efficacy subscales respectively, see Table 3) indicating a degree of coherence among the nurses on these subscales within each unit. This same degree of coherence was not seen in the emotional exhaustion subscale (ICC(1) = 0.032), and it was therefore entered as an individual level variable.

**Table 3:** Reliability of Data Aggregated Unit Level Variables

Variable	WMS	F	BMS	ICC(1)	ICC(2)	η2	ω^2^	PROB
MBI Cynicism	1.4412	7.6249	10.9893	0.2013	0.8688	0.2274	0.1974	0.0000

MBI Efficacy	0.9902	12.0921	11.9737	0.2968	0.9173	0.3174	0.2909	0.0000

Support for Innovative ideas	0.6401	3.6563	2.3405	0.0918	0.7265	0.1226	0.0890	0.0000

F = test statistic from one-way random effect ANOVA

The source table from ANOVA was used to calculate Interclass correlation 1 (ICC 1), Interclass correlation 2 (ICC 2), Eta Square (η^2^), and Omega Square (ω^2^) as follows:

1. ICC(1) = (BMS - WMS)/(BMS + [K - 1] WMS), where BMS is the between-group mean square, WMS is the within-group mean square, and K is the number of subjects per group. The average K for unequal group size was calculated as K = (1/[N - 1]) (ΣK - [ΣK^2 ^/ΣK]);

2. ICC(2) = (BMS - WMS)/BMS;

3. η2 = SSB/SST, where SSB is the sum of squares between groups and SST is the sum of squares total; and ω^2 ^= (SSB - [N - 1] WMS)/(SST + WMS).

4. ω^2 ^= (SSB - [N - 1] WMS)/(SST + WMS).

Experience on the unit, support for innovative ideas, and proportion of nurses possessing a baccalaureate degree or higher were collected using single items on the TROPIC survey. The remaining unit-level variables (specialty, language, and number of beds) were obtained as a result of the sampling strategy (in the case of specialty) or the TROPIC Unit Profile Form.

### Modeling process

A series of models was constructed for each of the 10 ACT concepts, resulting in 40 models for our analysis. First, an unconditional (null) model was run for each ACT concept (n = 10 models). The null model fits an overall constant to the data. It is equivalent to performing a random-effect analysis of variance that allows us to calculate how much of the variation in the 10 ACT (contextual) concepts lies between individuals and between units. This was then followed by a series of three models for each ACT concept (n = 30 models) as follows:

(1) Model 1 - a two-level model that fits the constant plus the individual-level variables selected for inclusion. As a result, Model 1 explains the proportion of the variance in each of the 10 contextual variables that is between individuals;

(2) Model 2 - a two-level model using individual variables and practice specialty (medical, surgical, critical care);

(3) Model 3 - a two-level model using individual and unit-level variables (including practice specialty). While practice specialty is a unit-level variable, we were interested in examining its unique contribution to variation because it was central to construction of the sampling frame for the study. Therefore we constructed Model 2 in addition to Model 3 to disentangle this contribution.

We started the modeling process with the construction of an unconditional or null model without any predictors specified at the individual or unit levels for each ACT concept. This allowed us to apportion the variance at the two levels. The null model was defined as:

Level 1:

Y_ij _= β_0j _+ ε_ij _, ε_ij _~ N(0, σ^2^) [Equation 1]

Level 2:

β_0j _= ψ_00 _+ ϑ_0j_, ϑ_0j _~ N(0, τ_00_) [Equation 2]

The combined null model is defined as:

Y_ij _= ψ_00 _+ ϑ_0j _+ ε_ij _[Equation 3]

Where:

Y_ij _= the value of the ACT (contextual) concept for the *i*^th ^nurse in the *j*^th ^unit

ψ_00 _= fixed term and represents the grand (or overall) mean score of the ACT (contextual) concept

ϑ_0j _= random term and represents unit offset effects from the grand mean or the discrepancy between overall mean and *j*^th ^unit mean (unique contribution of each patient care unit)

ε_ij _= random term and represents individual offset effects from the unit mean or individual's group mean (unique contribution of each individual *i *in patient care unit *j*)

Following examination of the 10 null models, an individual-level analysis was performed on each ACT contextual concept (Model 1 run 10 times). This allowed us to examine the predictive relationships between the individual-level independent variables and each ACT concept. Model 1 was defined as follows.

Model 1 (Level 1 and Level 2 Combined):

Y_ij _= ψ_00 _+ ϑ_0j _+ β_1 _(*employment status*)_ij_

+ β_2 _(*education*)_ij _+ β_3 _(age)_ij_

+ β_4 _*(burnout*-*emotional exhaustion*)_ij_

+ β_5 _(*adequate orientation*)_ij_

+ β_6 _(*job satisfaction*)_ij _+ ε_ij _[Equation 4]

Where:

Y_ij _= the value of the ACT (contextual) concept for the *i*^th ^nurse in the *j*^th ^unit

ψ_00 _= the overall average for the ACT (contextual) concept

β_1_, β_2_, β_3_, β_4_, β_5_, β_6 _= coefficients of the individual variables at Level 1

ε_ij _= the unique contribution of each individual *i *in patient care unit *j*

The errors, ε_ij_, are assumed independently, normally distributed with constant variance σ^2^. Since the control variables are centered on the sample means, the β_0j _is the mean achievement in a patient care unit after adjusting for the effect of employment status, education, age, burnout-emotional exhaustion, adequate orientation, and job satisfaction.

Model 1 was followed by the construction of Models 2 and 3, each of which were two-level models; Model 2 used individual variables and specialty as independent variables, while Model 3 used individual and unit-level variables (including specialty) as independent variables. Models 2 and 3 were defined as follows.

Model 2 (Level 1 and Level 2 Combined):

Y_ij _= ψ_00 _+ ψ_1 _(*specialty *)_j _+ β_1 _(*employment status*)_ij_

+ β_2 _(*education*)_ij _+ β_3 _(age)_ij_

+ β_4 _*(burnout*-*emotional exhaustion*)_ij_

+ β_5 _(*adequate orientation*)_ij_

+ β_6 _(*job satisfaction*)_ij _+ ε_ij _+ ϑ_j _[Equation 5]

Model 3 (Level 1 and Level 2 Combined):

Y_ij _= ψ_00 _+ ψ_1 _(*specialty*)_j_

+ ψ_2 _(*mean burnout-cynicism*)_j_

+ψ_3 _(*mean burnout-efficacy*)_j_

+ ψ_4 _(*mean years on unit*)_j_

+ ψ_5 _(*French-English status*)_j_

+ ψ_6 _(*mean number of unit beds*)_j_

+ ψ_7_(*mean support for innovative ideas*)_j_

+ ψ_8 _(% *baccalaureate or higher*)_j_

+ β_1 _(*employment status*)_ij _+ β_2 _(*education*)_ij_

+ β_3 _(*age*)_ij _+ β_4 _(*burnout-emotional exhaustion*)_ij_

+ β_5 _(adequate orientation)_ij_

+ β_6 _(job satisfaction)_ij _+ ε_ij _+ ϑ_j _[Equation 6]

Where:

Y_ij _= the value of the ACT (contextual) concept for the *i*^th ^nurse in the *j*^th ^unit

ψ_00 _= the overall average for the ACT (contextual) concept

ψ_1_, ψ_2_, ψ_3_, ψ_4_, ψ_5_, ψ_6 _, ψ_7_, ψ_8 _= the regression coefficients for the effect of unit level factors on the adjusted ACT (contextual) concept

β_1_, β_2_, β_3_, β_4_, β_5_, β_6 _= coefficients of the individual variables at Level 1

ε_ij _= the unique contribution of each individual *i *in patient care unit *j*

ϑ_j _= the unit level error term or the unique contribution of each unit to the unit level variation, τ. The ϑ_j's _are assumed to be normally distributed with variance, τ.

For all models, we assumed a random effect for the intercept and fixed effects for all of the Level 1 and Level 2 predictors. The variation between the 32 patient care units, or intraclass correlation (ICC), is the proportion of unconditional variance in each of the 10 dependent (contextual) variables attributable to the unit (i.e., before controlling for any individual background variables). ICC was calculated using the formula: ICC = τ_0_/(τ_0 _+ σ^2^) which is equivalent to the proportion of between-unit variance compared to the total variance in each of the 10 ACT concepts; where τ_0 _is the estimated unit-level error variance for the null model. The ICC measure was compared and assessed to determine whether unit-level variance was significantly different from 0. The relative reduction in unit-level error variance with respect to the null model (i.e., explained variance or *R^2^*) was subsequently assessed. For two-level multilevel models, the amount of variance explained between four models via the *R^2 ^*at Level 2 (the unit-level) can be calculated as R^2 ^= 1 - (τ_p_/τ_0_) where τ_p _is the estimated unit-level error variance for the model after *p *additional variables were added to the null model.

## Results

### Sample Characteristics

We analysed data from 844 professional nurses in 32 patient care units across 8 Canadian pediatric hospitals. The percentage distribution by practice specialty in the sample was balanced across the 8 hospitals: medicine (n = 14, 43.8%), surgery (n = 8, 25%), and critical care (n = 10, 31.2%). The number of occupied beds ranged from 4 to 46 with a mean of 20.04 beds (SD = 10.07 beds). This number was consistent across practice specialties with a mean of 20.76 beds (SD = 10.04), 21.68 beds (SD = 4.86), and 17.74 beds (SD = 13.28), for medicine, surgery, and critical care units respectively. The average length of patient stay was similar in medicine (6.41 days, SD = 2.99) and surgery units (4.34 days, SD = 1.11) and slightly higher (9.47 days, SD = 8.23) in critical care units. Descriptive statistics for each of the independent variables entered into the multilevel analysis are presented in Table 2. The aggregation analyses for the independent unit-level variables are presented in Table 3. Both Tables 2 and 3 report findings using a random effects ANOVA model and are descriptive and preliminary in nature to our main analysis, in which we used a series of multi-level (HLM) models. Variability of each of the dependent (ACT) variables is presented in Table 4 and findings from the multilevel analysis are in Tables 5, 6 and 7.

**Table 4:** Mean and Standard Deviation Scores on ACT Concepts by Unit and Specialty

Specialty	Unit	N	Leadership	Culture	**Eval**.	Formal Interact	Informal Interact	Social Capital	OS-Staff	OS-Space	OS-Time	Resources
**Surgical**	AA	32	3.39 (.85)	3.72 (.51)	2.80 (.70)	1.09 (.78)	5.07 (1.03)	3.85 (.36)	2.84 (1.10)	2.47 (.88)	2.80 (.60)	4.92 (1.67)
	
	CC	21	3.24 (.56)	3.68 (.70)	2.37 (.50)	1.79 (1.16)	4.21 (2.02)	3.59 (.48)	2.26 (.93)	2.37 (.87)	2.42 (.40)	4.19 (1.85)
	
	Z	26	2.92 (.81)	3.56 (.57)	2.08 (.79)	0.84 (.73)	4.40 (1.39)	3.62 (.53)	2.08 (1.06)	2.59 (1.01)	2.62 (.44)	3.12 (1.51)
	
	Q	20	3.06 (.91)	3.83 (.59)	2.96 (.83)	2.05 (.90)	5.50 (.79)	3.88 (.36)	2.95 (.90)	3.03 (.71)	3.05 (.29)	5.00 (1.79)
	
	D	15	3.39 (.69)	3.82 (.43)	2.67 (.99)	2.07 (.94)	5.29 (.83)	4.01 (.42)	2.40 (.87)	3.73 (.52)	2.63 (.54)	4.53 (1.33)
	
	BB	23	3.99 (.62)	4.19 (.58)	3.07 (.82)	1.93 (1.15)	5.50 (1.60)	4.03 (.44)	3.00 (1.17)	2.49 (1.11)	3.05 (.72)	5.27 (1.56)
	
	F	25	3.42 (.78)	3.75 (.53)	2.49 (.74)	0.92 (.77)	5.90 (1.26)	3.96 (.45)	2.70 (1.06)	2.79 (.88)	2.71 (.53)	4.00 (2.30)
	
	FF	37	3.70 (.76)	3.84 (.43)	2.95 (.76)	1.72 (.99)	5.46 (1.49)	3.88 (.38)	3.23 (.80)	3.48 (.57)	3.02 (.52)	5.77 (1.47)
	
	**Mean (SD)**		**3.41 (.82)**	**3.79 (.56)**	**2.69 (.81)**	**1.50 (1.03)**	**5.18 (1.45)**	**3.85 (.45)**	**2.73 (1.05)**	**2.86 (.94)**	**2.81 (.56)**	**4.67 (1.87)**

**Medical**	V	24	3.53 (.75)	3.69 (.60)	2.61 (.58)	0.98 (.97)	4.07 (1.47)	3.93 (.63)	3.15 (.98)	2.89 (.85)	2.76 (.61)	4.17 (1.92)
	
	J	19	3.66 (.64)	3.94 (.73)	2.76 (.89)	2.40 (.64)	5.21 (1.11)	4.05 (.38)	2.76 (.73)	3.37 (.67)	3.09 (.37)	5.22 (1.86)
	
	I	24	3.97 (.53)	3.71 (.35)	2.76 (.71)	1.30 (1.17)	3.52 (1.36)	3.83 (.41)	2.42 (.65)	1.87 (.80)	2.58 (.65)	3.72 (1.80)
	
	Y	17	3.54 (.75)	3.83 (.54)	2.98 (.52)	1.94 (1.03)	4.32 (1.85)	4.05 (.49)	2.38 (.86)	2.98 (.85)	2.68 (.54)	4.44 (2.02)
	
	T	32	3.78 (.39)	3.97 (.37)	3.29 (.72)	1.57 (.94)	5.23 (1.50)	3.94 (.48)	3.18 (.73)	2.85 (.77)	2.83 (.39)	4.69 (1.36)
	
	M	26	3.64 (.52)	4.04 (.36)	2.77 (.82)	1.77 (.95)	5.65 (1.42)	4.03 (.35)	3.35 (.96)	3.65 (.61)	2.97 (.59)	5.24 (1.95)
	
	C	35	3.89 (.62)	4.03 (.42)	3.34 (.58)	2.26 (1.00)	5.19 (1.39)	3.81 (.49)	2.84 (1.00)	3.29 (.85)	3.04 (.58)	5.37 (1.88)
	
	H	28	3.79 (.70)	3.96 (.35)	3.14 (.76)	1.91 (.88)	5.46 (1.15)	3.78 (.56)	2.95 (.93)	3.02 (.75)	2.80 (.47)	5.05 (1.66)
	
	S	20	3.51 (.57)	3.83 (.57)	2.86 (.69)	1.60 (.88)	4.98 (1.15)	3.88 (.28)	3.48 (.77)	2.65 (.91)	3.01 (.40)	4.37 (1.87)
	
	EE	19	3.85 (.99)	3.97 (.36)	2.73 (.75)	2.50 (1.07)	5.66 (1.53)	3.90 (.42)	4.40 (.49)	3.95 (.50)	3.59 (.61)	5.16 (1.62)
	
	W	20	4.19 (.44)	4.20 (.45)	3.18 (.86)	2.35 (1.30)	5.13 (1.69)	4.12 (.47)	2.85 (.92)	3.05 (.91)	3.05 (.50)	4.83 (2.01)
	
	K	22	3.21 (.81)	3.83 (.58)	2.58 (.72)	1.46 (1.14)	4.91 (1.45)	3.89 (.37)	3.25 (.75)	3.17 (.69)	2.85 (.42)	4.33 (1.86)
	
	B	23	3.96 (.50)	3.96 (.52)	2.91 (1.01)	1.91 (.96)	5.85 (1.67)	4.13 (.40)	3.57 (.79)	3.28 (.73)	3.04 (.41)	6.20 (1.51)
	
	E	20	4.03 (.79)	3.54 (.53)	2.61 (.61)	1.58 (1.09)	5.15 (1.66)	3.67 (.49)	1.93 (.75)	2.47 (.76)	2.66 (.69)	4.93 (1.82)
	
	**Mean (SD)**		**3.76 (.68)**	**3.90 (.50)**	**2.93 (.77)**	**1.81 (1.07)**	**5.04 (1.56)**	**3.92 (.47)**	**3.04 (.98)**	**3.04 (.89)**	**2.92 (.56)**	**4.86 (1.85)**

**Critical** **Care**	L	31	2.81 (.81)	3.61 (.59)	2.87 (.79)	2.02 (.85)	5.39 (1.18)	4.00 (.42)	3.44 (.90)	2.62 (.69)	3.23 (.59)	4.89 (1.88)
	
	X	30	3.67 (.66)	3.81 (.52)	2.45 (.65)	1.45 (.98)	4.10 (1.86)	4.01 (.44)	2.52 (.78)	2.79 (.85)	2.82 (.51)	3.98 (1.92)
	
	P	35	2.87 (.76)	3.52 (.57)	3.09 (.69)	1.84 (.88)	4.47 (1.63)	3.74 (.50)	2.30 (.91)	1.88 (.67)	2.71 (.62)	4.07 (1.68)
	
	N	30	3.91 (.60)	3.97 (.35)	3.47 (.75)	2.35 (.90)	5.82 (1.41)	4.01 (.44)	3.07 (.97)	3.06 (.69)	3.08 (.43)	5.90 (1.66)
	
	R	33	2.89 (.83)	3.71 (.52)	3.25 (.67)	1.73 (.98)	5.71 (1.92)	3.66 (.34)	3.41 (.99)	2.90 (.80)	2.96 (.58)	4.76 (1.77)
	
	U	30	4.08 (.65)	4.17 (.41)	2.88 (.71)	2.18 (.94)	5.35 (1.45)	4.19 (.59)	3.00 (.91)	2.44 (.76)	2.97 (.49)	3.75 (1.96)
	
	O	32	3.79 (.78)	3.91 (.50)	3.99 (.66)	2.38 (.79)	6.14 (1.56)	4.18 (.51)	1.89 (.69)	3.64 (.42)	2.92 (.49)	4.55 (1.57)
	
	DD	25	3.36 (.75)	3.57 (.42)	3.40 (.63)	2.42 (.75)	5.42 (1.54)	3.82 (.49)	2.40 (.102)	3.07 (.75)	3.15 (.61)	5.30 (1.80)
	
	A	59	3.28 (.81)	3.63 (.49)	2.92 (.77)	1.89 (.89)	5.09 (1.57)	3.75 (.41)	2.91 (.95)	2.70 (.81)	2.84 (.49)	4.99 (2.04)
	
	G	11	3.86 (.45)	3.73 (.51)	2.91 (.94)	2.36 (1.14)	5.50 (1.28)	3.82 (.39)	2.23 (.85)	2.70 (.78)	2.91 (.57)	3.77 (2.30)
	
	**Mean (SD)**		**3.40 (.86)**	**3.75 (.52)**	**3.12 (.81)**	**2.02 (.93)**	**5.27 (1.66)**	**3.91 (.49)**	**2.76 (1.02)**	**2.76 (.85)**	**2.95 (.55)**	**4.67 (1.93)**

**Overall mean (SD)**			**3.54 (.80)**	**3.82 (.53)**	**2.94 (.81)**	**1.81 (1.03)**	**5.16 (1.58)**	**3.90 (.47)**	**2.86 (1.02)**	**2.89 (.89)**	**2.90 (.56)**	**4.74 (1.89)**

P-value^1^			0.000	0.000	0.000	0.000	0.000	0.000	0.000	0.000	0.000	0.000

P-value^2^			0.000	0.001	0.000	0.000	0.199	0.191	0.000	0.000	0.018	0.368

^1 ^P-value for ANOVA to compare where there is mean difference on ACT concepts by unit or not.

^2 ^P-value for ANOVA to compare where there is mean difference on ACT concepts by specialty or not.

**Table 5:** Examination of Unit-Level Variation for the ACT Variables

Dependent Variable	Variance	Null	Model1^1^	Model2^2^	Model3^3^
**Leadership**	Variance component	0.1298**^4^	0.1205**	0.1036**	0.0797**
	ICC	0.2032	0.2073	0.1835	0.1473
	Explained variance (*R^2^*)	N/A	0.0716	0.2018	0.3863

**Culture**	Variance component	0.0257**	0.0165**	0.0154**	0.0003
	ICC	0.0928	0.0745	0.0697	0.0016
	Explained variance (*R^2^*)	N/A	0.3581	0.4021	0.9875

**Evaluation**	Variance component	0.1169**	0.1156**	0.0964**	0.0258*
	ICC	0.1770	0.1774	0.1524	0.0459
	Explained variance (*R^2^*)	N/A	0.0111	0.1754	0.7795

**Social Capital**	Variance component	0.0171**	0.0147**	0.0158**	0.0018
	ICC	0.0777	0.0716	0.0766	0.0095
	Explained variance (*R^2^*)	N/A	0.1437	0.0789	0.8940

**OS-Staffing**	Variance component	0.2555**	0.2042**	0.2004**	0.1698**
	ICC	0.2395	0.2199	0.2168	0.1900
	Explained variance (*R^2^*)	N/A	0.2008	0.2157	0.3354

**OS-Space**	Variance component	0.2136**	0.2078**	0.2105**	0.1104**
	ICC	0.2634	0.2709	0.2734	0.1648
	Explained variance (*R^2^*)	N/A	0.0272	0.0145	0.4831

**OS-Time**	Variance component	0.0371**	0.0304**	0.0300**	0.0169*
	ICC	0.1168	0.1050	0.1039	0.0615
	Explained variance (*R^2^*)	N/A	0.1807	0.1905	0.5430

**Formal** **Interactions**	Variance component	0.1730**	0.1375**	0.1145**	0.0963**
	ICC	0.161	0.1386	0.1181	0.1012
	Explained variance (*R^2^*)	N/A	0.2052	0.3382	0.4435

**Informal** **Interactions**	Variance component	0.2886**	0.2180**	0.2287**	0.0710
	ICC	0.1155	0.0938	0.0979	0.0326
	Explained variance (*R^2^*)	N/A	0.2446	0.2076	0.7541

**Structural and electronic** **resources**	Variance component	0.3490**	0.2840**	0.3059**	0.1655*
	ICC	0.0979	0.0831	0.0890	0.0502
	Explained variance (*R^2^*)	N/A	0.9169	0.9110	0.9498

^1 ^Model 1: HLM with individual level covariates only

^2 ^Model 2: HLM with individual level covariates + specialty

^3 ^Model 3: HLM with both individual + specialty + unit level covariates

^4 ^Test whether unit error variance is greater than 0 (*: significant at 0.05 level, **: significant at 0.01 level)

**Table 6:** Contribution of Individual, Specialty, and Unit-Level Variables using *R^2^*

Dependent Variable	Explained variance (*R^2^*)			
	
	Null vs. Model 1^1^	Model 1 vs. Model 2^2^	Model 1 vs. Model 3^3^	Model 2 vs. Model 3^4^
**Leadership**	7.16%	14.02%	33.89%	23.11%

**Culture**	35.81%	6.85%	98.05%	97.90%

**Evaluation**	1.11%	16.62%	77.70%	73.25%

**Social Capital**	14.37%	-7.57%	87.62%	88.49%

**OS-Staffing**	20.08%	1.86%	16.85%	15.27%

**OS-Space**	2.72%	-1.30%	46.87%	47.55%

**OS-Time**	18.07%	1.19%	44.22%	43.55%

**Formal interactions**	20.52%	16.73%	29.98%	15.91%

**Informal** **interactions**	24.46%	-4.91%	67.44%	68.96%

**Structural and** **electronic resources**	91.69%	-7.71%	41.73%	45.90%

^1 ^*R^2 ^*= 1 - (τ_1_/τ_0_) where τ_0 _and τ_1 _is the estimated unit-level error variance for the null model and model 1. This measure implies the contribution of individual level covariates in terms of relative error variance reduction when individual level covariates were added to null model.

^2 ^*R^2 ^*= 1 - (τ_2_/τ_1_) where τ_1 _and τ_2 _is the estimated unit-level error variance for models 1 and 2. This measure implies the contribution of specialty in terms of relative error variance reduction when specialty was added to model 1.

^3 ^*R*^2 ^= 1 - (τ_3_/τ_1_) where τ_1 _and τ_3 _is the estimated unit-level error variance for the model 1 and 3. This measure implies the contribution of specialty and unit level relative covariates in terms of error variance reduction when specialty and unit level covariates were added to model 1.

^4 ^*R^2 ^*= 1 - (τ_3_/τ_2_) where τ_2 _and τ_3 _is the estimated unit-level error variance for the model 2 and 3. This measure implies the contribution of specialty and unit-level relative covariates in terms of error variance reduction when unit-level covariates were added to model 2.

Note: negative *R^2 ^*reported in-text as '0'

**Table 7:** Significant Explanatory Variables on each ACT Concept

Dependent Variable	Level	Model 3 (individual, specialty and unit variables)	Coefficients	SE	P-value
**Leadership**	**Individual Unit**	Exhaustion	-0.6610	0.0217	0.0024
		Job satisfaction	0.1925	0.0362	< 0.0001
		Support for innovative ideas	0.6610	0.2394	0.0114

**Culture**	**Individual Unit**	Education (Diploma)^1^	0.3123	0.1313	0.0224
		Education (Bachelor)	0.2797	0.1288	0.0360
		Age	-0.0337	0.0092	0.0003
		Exhaustion	-0.0432	0.0144	0.0028
		Adequate orientation	0.0639	0.0222	0.0042
		Job satisfaction	0.2169	0.0236	< 0.0001
		Cynicism	0.1794	0.0681	0.0151
		Years on unit	0.0196	0.0089	0.0392
		Support for innovative ideas	0.4343	0.0675	< 0.0001

**Evaluation**	**Individual Unit**	Employment status (part time)^2^	-0.2879	0.1404	0.0458
		Specialty (surgical)^3^	-0.6672	0.1181	< 0.0001
		Specialty (medical)	-0.4444	0.1223	0.0015
		Unit size (no. beds)	0.0128	0.0035	0.0014
		Support for innovative ideas	0.9394	0.1644	< 0.0001
		Proportion of baccalaureate or higher	-0.0095	0.0043	0.0393

**Social capital**	**Individual Unit**	Adequate orientation	0.0708	0.0214	0.0010
		Job satisfaction	0.1029	0.0229	< 0.0001
		Unit size (no. of beds)	-0.0044	0.0015	0.0102
		Support for innovative ideas	0.3763	0.0719	< 0.0001

**OS - Staffing**	**Individual Unit**	ExhaustionJob satisfactionN/A	-0.15170.2027N/A	0.02730.0454N/A	< 0.0001 < 0.0001N/A

**OS - Space**	**Individual Unit**	Exhaustion	-0.0819	0.0240	0.0007
		Job satisfaction	0.1126	0.0400	0.0050
		Years on unit	0.0737	0.0343	0.0436
		Support for innovative ideas	0.7787	0.2788	0.0106

**OS - Time**	**Individual Unit**	Exhaustion	-0.0809	0.0162	< 0.0001
		Job satisfaction	0.1241	0.0269	< 0.0001
		N/A	N/A	N/A	N/A

**Formal interactions**	**Individual Unit**	Exhaustion	-0.1074	0.0297	0.0003
		Specialty (surgical)	-0.5417	0.1975	0.0119
		Support for innovative ideas	-0.3962	0.2759	0.0226

**Informal interactions**	**Individual Unit**	Exhaustion	-0.1290	0.0464	0.0056
		Support for innovative ideas	0.8710	0.2970	0.0077

**Structural and electronic resources**	**Individual Unit**	Exhaustion	-0.1500	0.0568	0.0084
		Support for innovative ideas	0.9376	0.4087	0.0317

^1 ^Reference group for Education: Master or higher

^2 ^Reference group for Employment status: Casual

^3 ^Reference group for Specialty: Critical Care

### Reliability of Aggregated Unit-Level Variables

The statistics to assess the reliability of aggregated values supported aggregating the data on these variables to the level of the patient care unit (Table 3). Statistically significant (p < 0.05) *F *statistics and/or ICC(2) values greater than 0.60 indicate greater reliability and justification for aggregating the variables to the unit-level. The ICC(1) values ranged from 0.0918 to 0.2968, indicating perceptual agreement among nurses about the mean values for the variables within each unit. That is, the nurses' perceptions about their own unit were similar. The relative effect sizes for both η^2 ^and ω^2 ^values were moderate, suggesting that, as we aggregated data, our ability to assign the same meaning for a variable at the unit-level that we had at the individual-level decreased.

### Variability in the Dependent Variables

**Figure 1:**
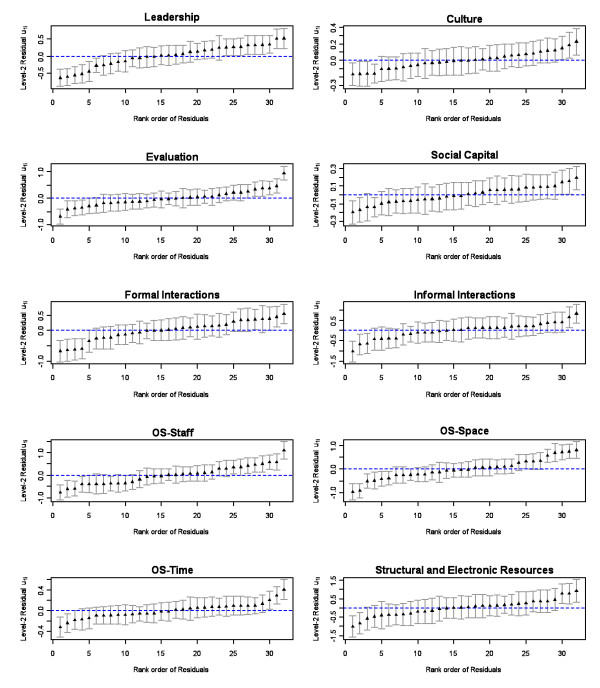
**Caterpillar Plot for each ACT Variable (Model 1, N = 32 Units)**.

To assess variation in the dependent variables (the 10 ACT contextual concepts) examined in this study, we: (1) examined the mean scores for each concept by unit and by specialty (Table 2), and (2) constructed a series of caterpillar plots (Figure 1) examining the 10 dependent variables across the full sample of 32 patient care units. There were statistically significant differences between mean scores on all 10 dependent variables by unit (ANOVA, p < 0.001, Table 2) and for 7 of the 10 dependent variables (exceptions were informal interactions, social capital, and structural and electronic resources) by practice specialty (ANOVA, p < 0.05, Table 2). The caterpillar plots (Figure 1) were generated using the null hierarchical linear models and 95% confidence intervals; the MLwiN 2.12 program was used to generate these plots. The ascending order of mean scores seen in the caterpillar plots indicate that some units departed significantly from the overall level of each of the 10 ACT concepts across the full sample. These findings demonstrate adequate variability in the dependent variables.

### Results of the Multi-Level (HLM) Analysis

#### Null model

The components of separate variances at the two levels (individual and unit) varied by the dependent ACT concept variable: Level 1 individual variance ranged from 0.2031 to 3.2173 (p < 0.001) and Level 2 unit variance ranged from 0.0171 to 0.3490 (p < 0.001). Each was statistically significant at the 0.01 level. These variance components were then used to estimate the ICC at the unit level. This proportion varied according to the dependent variable (ACT concept) as follows: leadership (0.2032), culture (0.0928), evaluation (0.1770), social capital (0.0777), organizational slack-staffing (0.2395), organizational slack-space (0.2634), organizational slack-time (0.1168), formal interactions (0.0161), informal interactions (0.1155), and structural and electronic resources (0.0979). Each was statistically significant at the 0.01 level (Table 5).

#### Analysis of individual predictors (Model 1)

Findings revealed that the contribution of individual-level variables in terms of relative error variance reduction when they were added into each null model (i.e., for each of the 10 contextual variables) varied significantly according to the ACT concept examined, ranging from a low of 0.0111 (evaluation) to a high of 0.9169 (structural and electronic resources) (Table 5 Column 4). The proportions of explained variance (*R^2^*) for all 10 ACT concepts across the three models are presented in Table 5.

#### Analysis of individual and specialty predictors (Model 2)

We had hypothesized that part of the variance in the 10 ACT concepts should reflect practice specialty (medicine, surgery, and critical care). In Model 2, we assessed for the effect of unit specialty on between-unit variation. Practice specialty accounted for, from 0% (for four contextual variables: social capital, organizational slack-staff, informal interactions, and structural and electronic interactions) to almost 17% (for two contextual variables: evaluation [0.1662] and formal interactions [0.1673]) of the variance (Table 6 column 3: Model 1 vs. Model 2). This proportion of explained variance is after controlling for individual-level variables but prior to controlling for other unit-level variables.

#### Analysis of individual and specialty and other unit predictors (Model 3)

In Model 3, seven additional unit-level variables were added to the model (Table 2). The unique contribution of these unit-level variables to explaining variance in each of the 10 ACT concepts (i.e., after controlling for individual-level variables and practice specialty) is summarized in Table 6 (see column 5: Model 2 vs. Model 3).

The test for unit-level variance was significant for 7 of the 10 ACT concepts. The seven unit-level variables in Model 3 combined accounted for between 0.1527 and 0.7325 of the variations as follows: leadership (0.2311), evaluation (0.7325), organizational slack-staffing (0.1527), organizational slack-space (0.4755), organizational slack-time (0.4355), formal interactions (0.1591), and structural and electronic resources (0.4590). Model 3 results also indicate that significant residual (unexplained) variations remained after controlling for individual and unit-level variables entered into our models. For example, less than 60% of the variance was explained in the following five contextual variables: (1) leadership (0.3863 explained variance), (2) organizational slack-staffing (0.3354 explained variance), (3) organizational slack-space (0.4831 explained variance), (4) organizational slack-time (0.5430 explained variance), and (5) formal interactions (0.4435 explained variance) (Table 5 column 6: Model 3).

Finally, we assessed which unit-level variables were associated, at statistically significant levels, with each of the 10 ACT concepts in our multilevel analysis (Table 7). 'Support for innovative ideas' was the only unit-level variable that showed a consistent, statistically significant association across the majority (n = 8 of 10) of ACT concepts; the two exceptions were organizational slack-staffing and organizational slack - time. Specialty showed an influence that was statistically significant on two of the contextual variables: evaluation and formal interactions. When compared to critical care, both surgical (0.66, p < 0.001), and medical (-0.44, p < 0.001) units had lower scores on evaluation that were statistically significant. Surgical units (-0.54, p = 0.011) had statistically significant lower scores on formal interactions compared to both medicine and critical care units. Other unit-level variables associated, at statistically significant levels, with the contextual variables in our multilevel analysis included:

• burnout-cynicism (with culture)

• years on the unit (experience) (with culture and organizational slack-space)

• unit size (with evaluation and social capital)

• percentage of baccalaureate or higher prepared nurses (with evaluation)

## Discussion

The findings reported here add to the validity evidence supporting the use of the ACT to discriminate patient care units by all 10 ACT contextual factors. In addition, we found evidence of relationships between a variety of individual- and unit-level variables that explained much of this between-unit variation for each of the 10 ACT concepts.

### Aggregation of the ACT Concepts

The aggregation statistics performed in this study support the argument that ACT responses obtained from pediatric nurses (in our study sample) can be aggregated reliably and validly to obtain unit-level estimates of the dimensions of context represented in the ACT. This is consistent with our findings in the context of healthcare aides' scores in long-term care settings [19]. We ran the same aggregation statistics on allied healthcare professionals (e.g., rehabilitation therapists) (n = 209, mean = 7 responses/unit) who also competed the ACT survey in the study reported in this paper. These aggregation statistics did not support aggregation at the unit level. This is consistent with allied healthcare professionals' work practices being more aligned with programs (which consist of several units) rather than a single unit (where most nurses tend to work). The remaining respondent groups from the study were small in number (physicians n = 86, mean = 3 responses/unit; practice specialists n = 55, mean = 2 responses/unit; and managers n = 35, mean = 1 response/unit) and therefore we did not perform unit-level aggregation statistics on their responses. We suspect, however, that similar to allied healthcare professionals, their responses would align more with programs or possibly facilities (depending on their context of care delivery) rather than the unit.

### Discrimination Between Patient Care Units

Our first objective was to examine the extent to which the 10 ACT concepts discriminate between patient care units. The majority of patient care is delivered within microsystems (i.e., within patient care units). The microsystems literature, according to Disch [22], highlights the importance of focusing on the unit, rather than the individual, as the unit of analysis. As such, work in this field has concentrated on understanding the context of care delivery and the optimization of systems to enable health professionals to deliver high quality care. Research evidence indicates that development of best practice within microsystems has the potential to improve patient outcomes [21]. Contextual variation at the unit level in healthcare using validated instruments has been largely unexplored. However, a recent study of public health and social services settings in Finland examined differentiation in organizational culture and climate across work units [42]. Individual-level data were collected using the Organizational Social Context (OSC) instrument [43] to measure work unit culture and climate. The investigators concluded that different organizational climates and cultures exist within work units and at organizational levels. Given the importance of the patient care unit as an essential functional component of an organization (one at which quality of care and patient safety are realized) [21,22,44], the capacity of the ACT to discriminate between such units is a highly desirable feature of the instrument.

To assess variation in the 10 ACT concepts as dependent variables, we assessed the mean scores for each concept by unit and by practice specialty. The statistically significant differences, between mean scores on all 10 concepts by unit and for 9 of the 10 concepts by practice specialty (Table 4) and the ascending order of mean scores in the caterpillar plots (Figure 1), show that some units departed significantly from the overall level of each of the 10 concepts across the sample. These findings suggest adequate variability between units on the ACT concepts in this sample. Such findings, therefore, provide evidence for the capacity of the ACT to discriminate between units. This attribute of the instrument is vital to distinguishing and measuring contextual dimensions of the patient care functional unit that are important to optimizing quality of care. This instrument, therefore, shows promise in offering a measure of the status of the microsystem and highlighting areas in which modifications are required.

A recent comparative analysis of measurement tools for organizational context demonstrates some overlap with extant context tools and the 10 dependent variables in ACT. In this analysis, French and colleagues [45] identified 18 tools; the ACT was not included due to the date restrictions of their study. Seven common themes or attributes across the 18 tools were identified: organizational learning culture, vision, leadership, knowledge need, acquisition of new knowledge, knowledge sharing, and knowledge use. Four of these themes are conceptually similar to the ACT concepts, specifically organizational learning culture (with ACT culture), leadership (with ACT leadership), knowledge sharing (with several ACT concepts including formal interactions, informal interactions, organizational slack-time) and knowledge use (with ACT formal interactions and ACT informal interactions). Eleven of the eighteen tools identified by French and colleagues [45] contained elements of these four themes. The majority of these tools (8 of 11) were developed in the field of organizational theory generally, and were not specific to healthcare. Three tools had some conceptual similarity to ACT concepts: (1) the ABC Survey [46] (attributes assessed: knowledge sharing, knowledge use); (2) KEYS Knowledge Exchange Yields Success Questionnaire [47] (attribute assessed: leadership); and, (3) the Research and Development Index [48] (attribute assessed: knowledge use). Two of these three tools (ABC Survey and KEYS Knowledge Exchange Yields Success Questionnaire) do not have published reliability and validity assessments and the third tool (Research and Development Index) has only been used at an organizational (NHS Trust) level, not at a unit level.

### Discrimination Between Specialties

Previous multivariate research by Mallidou et al. [49] demonstrated the existence of nurse specialty subcultures. In that research, four nursing specialty cultures were assessed: (1) medical, (2) surgical, (3) intensive care, and (4) emergency care. Mallidou and colleagues demonstrated that nurse and patient outcomes (e.g., job satisfaction, quality of care and adverse patient occurrence) in acute care hospitals were shaped by nursing specialty subcultures. In our research, while *practice specialty *contributed independently to the explained variance, it is less clear whether our findings support its inclusion as a sampling criterion. For instance, in four of the 10 ACT concepts (social capital, organizational slack-staffing, informal interactions, and structural and electronic interactions) practice specialty accounted for 0% of the variance; while in two concepts, it accounted for almost 17% of the variance (evaluation and formal interactions). Specialty only showed a statistically significant association with two of the contextual concepts - evaluation and formal interactions, with critical care respondents scoring higher in both cases.

Upon further reflection of our findings in relation to Mallidou et al.'s [49] study, a conceptual issue and an inter-related unit of analysis issue become apparent; that is, what is the appropriate scope of a specialty? Said another way, it could be argued that in the case of this research, only one practice specialty was explored, that is, pediatrics - and further categorizing of nurses into medical, surgical, and critical care is more accurately a sub-specialty classification. That said, the scope and extent of practice specialty and potentially sub-specialty sampling criteria demand careful consideration of how nurses ascribe membership to particular practice specialties of nursing, and as a result, this methodological decision must be thoughtfully weighed by investigators.

*Support for innovative ideas *was the only unit-level variable that showed a consistent and statistically significant association with the majority (8 of 10) of ACT context variables; the two exceptions were two of the organizational slack concepts (staffing and time). Underpinning these findings is an assumption that support for innovativeness is a collectively held value and that support for innovation behaves in a manner over and above the additive behavior of the individual members in the unit. These findings parallel some of the ideas originally put forth by Rogers [50] who suggested that innovativeness is related to variables such as leadership, internal organizational structural characteristics and external characteristics of the organization. Several of the ACT concepts map onto Rogers' ideas, for instance, the ACT concept of leadership maps onto leadership, and formal and informal interactions map onto internal organizational structural characteristics. The strong association between support for innovative ideas and eight of the 10 ACT contextual variables suggests the importance of support for innovative ideas in explaining the between-unit variation for the concept, particularly given that individual background and practice specialty factors were controlled for in our models.

In our final model results (Model 3), we can see that significant residual unit variations remain after controlling for the individual and the unit-level variables entered into our models. Less than 60% of the variance was explained in leadership, organizational slack-staffing, organizational slack-space, organizational slack-time, and formal interactions. This suggests that future research is needed to identify other factors that may help explain the residual variation remaining in these contextual variables.

### Limitations

We might have explored further Level 1 regression equations that model each of the within- patient care unit regression coefficients as a function of the unit-level factors if the slopes were allowed to vary among the units (i.e., a random-effect models). However, we deemed the sample size per unit (on average 25 nurses) too small to explore cross-level interaction, making it impossible to estimate the variability in such regression coefficients accurately. Therefore all regression coefficients other than the intercept were constrained to be constant within units (i.e., a fixed-effect model).

## Conclusion

The findings reported here represent the third published argument for validity of the ACT and add to the evidence supporting its use to discriminate patient care units by all 10 contextual concepts. We further found evidence of relationships between a variety of individual- and unit-level variables that explained much of this between-unit variation for each of the 10 ACT concepts. Future research will include an examination of the relationships between the ACT's contextual factors and research utilization by nurses and ultimately the relationships between context (as measured by the ACT), research utilization, and outcomes for patients.

## Competing interests

The authors declare that they have no competing interests.

## Authors' contributions

CAE, SS, GGC and BS participated in designing the study and securing its funding. CAE, GGC, SHK, and WKM designed the analytic plan for the analyses presented in this paper. SHK conducted the statistical analysis; CAE, JES, SHK, and WKM interpreted the statistical analysis. CAE, JES, AMH, SS, GGC, SHK and WKM participated in drafting the manuscript. All authors provided critical commentary on the manuscript and approved the final version.

## Pre-publication history

The pre-publication history for this paper can be accessed here:

http://www.biomedcentral.com/1472-6963/11/251/prepub

## Supplementary Material

Additional File 1 **Inclusion and Exclusion Criteria by Professional Group**. A summary of the inclusion and exclusion criteria applied to healthcare professionals in the study. Click here for file

Additional File 2 **Intraclass Correlation Calculation**. Compares intraclass correlation calculated using random coefficient (multi-level) model and one-way random-effects ANOVA models. Click here for file
