# Supplementary material for: Assessment of variation in the alberta context tool: the contribution of unit level contextual factors and specialty in Canadian pediatric acute care settings
Source: BMC Health Serv Res. 2011 Oct 4;11:251. doi: 10.1186/1472-6963-11-251 (PMC3200177; doi:10.1186/1472-6963-11-251)
Supplement: Additional File 2 — Intraclass Correlation Calculation. Compares intraclass correlation calculated using random coefficient (multi-level) model and one-way random-effects ANOVA models. [file 1472-6963-11-251-S2.DOC]

**Additional File 2. Calculation of Intraclass Correlation**

Approaches employed

(1) Random coefficient (multi-level) models - calculated as ICC = unit-level variance/ (unit-level variance + individual level variance)

(2) One-way random-effects ANOVA model – calculated as ICC(1) = (BMS – WMS)/(BMS + [K-1] WMS) where BMS = between mean square, WMS = within mean square, and K= the number of participants per group.

Note: the ICC(1) can also be expressed as following,ICC(1) = (BMS-WMS)/(BMS+[k-1]WMS) = τ_00_ / (τ_00_ + σ^2^) = unit-level variance / (unit-level variance + individual-level variance), where τ_00_ (unit-level variance)=(BMS-WMS)/k, σ^2^ (individual-level variance)=WMS

**Example 1 (variables in Table 3 in manuscript)**

| Variable | ICC from random-effect ANOVA | ICC from HLM |
| --- | --- | --- |
| MBI Cynicism | 0.2013 | 0.2172 |
| MBI Efficacy | 0.2968 | 0.3165 |
| Support for Innovative ideas | 0.0918 | 0.0856 |

**Example 2 (variables in Table 5 in manuscript)**

| Variable | ICC from random-effect ANOVA | ICC from HLM  (Null model) |
| --- | --- | --- |
| Leadership | 0.2091 | 0.2032 |
| Culture | 0.0940 | 0.0928 |
| Evaluation | 0.1819 | 0.1770 |
| Social capital | 0.0779 | 0.0777 |
| OS-staff | 0.2225 | 0.2395 |
| OS-space | 0.2591 | 0.2634 |
| OS-time | 0.1062 | 0.1168 |
| Formal Interactions | 0.1539 | 0.1610 |
| Informal interactions | 0.1116 | 0.1155 |
| Structural and electronic resources | 0.0964 | 0.0975 |
